# Prevalence of Allergic Bronchopulmonary Aspergillosis in Bronchial Asthma Patients: A Retrospective Observational Study

**DOI:** 10.7759/cureus.90863

**Published:** 2025-08-24

**Authors:** Arnab Swain

**Affiliations:** 1 Pulmonary Medicine, Postgraduate Institute of Medical Education and Research and Capital Hospital, Bhubaneswar, IND

**Keywords:** abpa, aspergillus fumigatus, bronchial asthma, hrct, ige, pft

## Abstract

Background

Asthma is a chronic inflammatory airway disease that can be exacerbated by fungal allergens, particularly *Aspergillus fumigatus*. Allergic bronchopulmonary aspergillosis (ABPA) is a severe hypersensitivity reaction observed in a subset of asthma patients.

Objective

To determine the prevalence of ABPA in patients with bronchial asthma and to describe their clinical, radiological, and immunological profiles.

Methods

This retrospective observational study was conducted over 18 months at a tertiary care hospital. Data from 100 bronchial asthma patients undergoing pulmonary function testing (PFT) were reviewed. ABPA diagnosis was based on criteria including elevated serum total IgE, specific IgE to *A. fumigatus*, chest X-ray findings, पigh-resolution computed tomography (HRCT), and eosinophilia.

Results

ABPA was diagnosed in 14% of asthma patients. The most common age group was 21-30 years. Male predominance (69%) was noted. Dyspnea (64%) was the most frequent symptom. ABPA was observed in 21% of mild, 7% of moderate, and 11% of severe asthma cases. Chest infiltrates had 100% sensitivity but low specificity (44.19%) for ABPA diagnosis.

Conclusion

ABPA is present in a significant proportion of asthma patients. Early identification is crucial due to treatment implications. Chest imaging and serum IgE levels are useful screening tools.

## Introduction

Asthma is a chronic inflammatory disorder of the airways marked by persistent inflammation, progressive decline in pulmonary function, and structural remodeling of airway tissues. It affects individuals across all age groups and, when poorly controlled, can significantly impair daily activities and may even lead to fatal outcomes [1]. Over the past few decades, the global prevalence of asthma has increased to near-epidemic levels and is projected to rise substantially over the next 15 to 20 years [2]. Clinically, asthma is characterized by symptoms such as breathlessness, wheezing, and variable airflow obstruction.

Environmental exposure to fungal spores has been recognized as a potential trigger for worsening asthma symptoms and lung function. Fungi may contribute to severe asthma in several ways: (a) through direct inhalation of fungal spores, (b) through fungal sensitization, which may or may not be associated with severe asthma, and manifests as immediate hypersensitivity reactions or elevated fungus-specific IgE levels, and (c) through the development of allergic bronchopulmonary mycosis (ABPM), a severe form of sensitization resulting in irreversible bronchopulmonary damage. Approximately 112 genera of fungi are known to act as allergen sources, with *Aspergillus*, *Alternaria*, *Cladosporium*, and *Penicillium *being the most common [3].

Among these, *Aspergillus fumigatus *is the most frequently implicated species. It colonizes the lower respiratory tract and serves both as an allergen and a pathogen [4]. Fungal asthma exists on a clinical spectrum, ranging from mild *A. fumigatus *sensitization to more severe forms such as allergic bronchopulmonary aspergillosis (ABPA). ABPA is characterized by asthma exacerbations, recurrent pulmonary infiltrates, central bronchiectasis, elevated total serum IgE levels, increased *A. fumigatus*-specific IgE or IgG, eosinophilia, and the production of mucus plugs. According to recent diagnostic criteria [5], ABPA is identified by the presence of asthma, *A. fumigatus*-specific IgE ≥ 0.35 kUA/L, total IgE > 1000 IU/mL, and *A. fumigatus*-specific IgG ≥ 27 mgA/L. ABPA is further classified into serological ABPA (ABPA-S), where high-resolution computed tomography (HRCT) chest findings are normal, and ABPA with bronchiectasis (ABPA-B), where bronchiectasis is evident.

Distinguishing between these conditions is critical, as management differs: patients with fungal sensitization typically require observation alone, while those with ABPA require targeted treatment. In light of these considerations, the present study was conducted to determine the prevalence of ABPA among patients with bronchial asthma and to evaluate their clinical and radiological profiles.

## Materials and methods

Study design and setting

This retrospective observational study was conducted in the Department of Pulmonology at a tertiary care teaching hospital in western India, over a period of 18 months (November 2015 to May 2017). A total of 100 patients with a confirmed diagnosis of bronchial asthma were included. Inclusion criteria were: patients of any age and sex with a clinically confirmed diagnosis of bronchial asthma based on clinical evaluation and pulmonary function testing (PFT). Patients attending either the outpatient department (OPD) or admitted to the inpatient department (IPD) during the study period. No exclusion criteria were applied.

*Asthma Severity Classification* 

Asthma severity was classified as mild, moderate, or severe according to the Global Initiative for Asthma (GINA) guidelines described by Reddel HK et al., based on clinical symptoms and spirometric findings [6].

Diagnostic Criteria for ABPA

ABPA was diagnosed using a combination of clinical, immunological, and radiological parameters in line with established criteria, as mentioned in Agarwal et al. [2].

Pulmonary Function Test (PFT)

Spirometry with bronchodilator reversibility was defined as an increase in forced expiratory volume in one second (FEV₁) of ≥12% and ≥200 mL after administration of 200 μg of inhaled salbutamol, according to the American Thoracic Society (ATS) guidelines described by Miller MR et al. [7].

Serum Total IgE

Serum total IgE was measured using ELISA (Roche Diagnostics Corporation, Indianapolis, USA; calibration performed before each batch run). A level >1000 IU/mL was considered suggestive of ABPA.

*Specific IgE to *Aspergillus fumigatus*:*

Specific IgE to *Aspergillus fumigatus *was measured using Roche's immunoassay, with values ≥0.35 kUA/L considered positive.

Peripheral Blood Eosinophilia

Peripheral blood eosinophilia was defined as an absolute eosinophil count greater than 500 cells/µL.

Chest Radiography (X-ray)

Films were reviewed for characteristic features such as fleeting pulmonary infiltrates, tramline shadows, toothpaste/gloved-finger opacities, and fibrotic changes.

High-Resolution Computed Tomography of the Thorax

HRCT was performed using a standardized protocol with 1-1.5 mm slice thickness. Findings such as central bronchiectasis, mucus plugging, and fibrotic changes were noted.

Radiological Interpretation and Observer Variability

All chest X-rays and HRCT images were independently reviewed by two experienced radiologists who were blinded to clinical and serological data. Discrepancies were resolved by consensus discussion.

Data Collection and Case Ascertainment

Data were extracted from patient records, including demographic details, clinical history, comorbidities, laboratory results, and imaging findings. All patients meeting the inclusion criteria during the study period were included, reducing the risk of selection bias.

Statistical analysis

All collected data were entered into Microsoft Excel (Microsoft Corporation, Redmond, USA) and analyzed using SPSS version 22.0 (IBM Corp., Armonk, USA). Categorical variables were expressed as frequencies and percentages. Associations between categorical variables were tested using the chi-square test. A p-value <0.05 was considered statistically significant. 

## Results

Patients and disease characteristics

A total of 100 patients diagnosed with bronchial asthma who attended the Outpatient Department (OPD) or the Inpatient Department (IPD) of a tertiary care hospital between November 2015 and May 2017 were retrospectively included in the study. The mean age of the study population was 30.45 years. The most common age group was 21 to 30 years (27%), followed closely by 18 to 20 years (26%), 31 to 40 years (23%), and 51 to 60 years (14%) (Table 1).

**Table 1:** Study Population The most common age group amongst the study population was 21 to 30 years (27%), followed by 18 to 20 years (26%), 31 to 40 years (23%), and 51 to 60 years (14%).

Age group	Frequency	Percent
18 to 20 years	26	26
21 to 30 years	27	27
31 to 40 years	23	23
41 to 50 years	3	3
51 to 60 years	14	14
more than 60 years	7	7
Total	100	100

**Figure 1:**
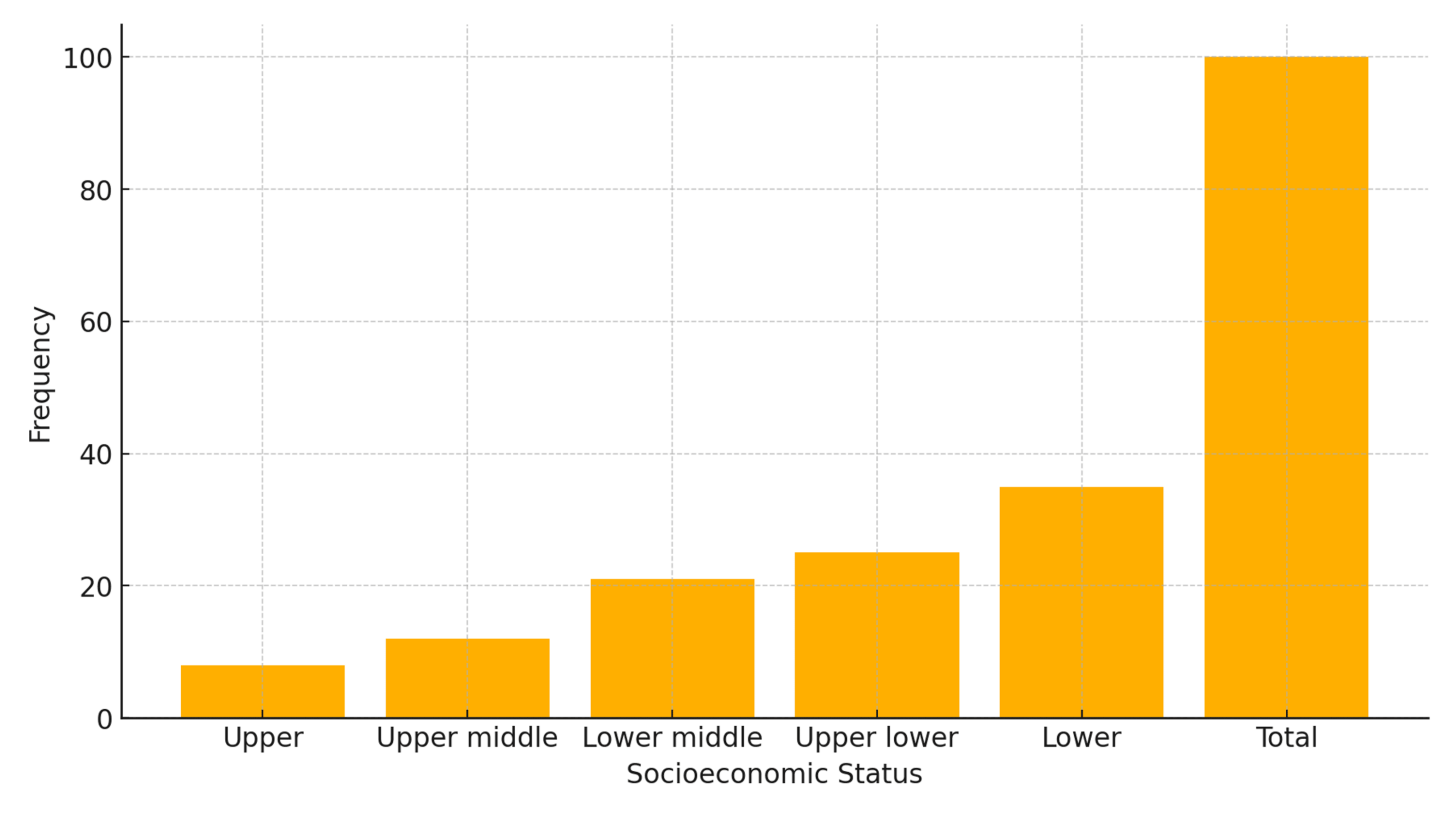
Socioeconomic Status Most of the study population belonged to the lower socioeconomic status (36%), followed by upper-lower (24%), lower-middle (21%), and upper-middle (12%) groups.

There was a marked male predominance, with 69% male and 31% female participants. In terms of socioeconomic distribution, most patients belonged to the lower socioeconomic strata (36%), followed by upper-lower (24%), lower-middle (21%), and upper-middle classes (12%) (Figure 1).

Asthma severity was assessed and found to be mild in 48% of cases, moderate in 43%, and severe in 9% (Table 2).

**Table 2:** Severity of Asthma vs ABPA Allergic bronchopulmonary aspergillosis (ABPA) was observed in 21% of mild asthma, 7% of moderate asthma, and 11% of severe asthma cases, and the difference was statistically insignificant (*p* = 0.49).  Data presented as numbers and percentages: n (%).

Severity of asthma	ABPA		Total
	Present	Absent	
Mild	10 (21%)	38 (79%)	48 (100%)
Moderate	3 (7%)	40 (93%)	43 (100%)
Severe	1 (11%)	8 (89%)	9 (100%)
Total	14 (14%)	86 (86%)	100 (100%)

Radiological and diagnostic evaluations

Chest X-ray infiltrates were observed in 62% of patients. Among ABPA patients, chest infiltrates were present in all cases, though specificity was low (Table 3).

**Table 3:** ABPA vs Chest X-Ray Infiltrates Chest X-ray Infiltrates were observed in 23% of allergic bronchopulmonary aspergillosis (ABPA) cases. The association was statistically significant (p = 0.001). Data are presented as numbers and percentages: n (%).

Chest X-ray Infiltrates	ABPA		Total
	Present	Absent	
Yes	14 (23%)	48 (77%)	62 (100%)
No	0 (0%)	38 (100%)	38 (100%)
Total	14 (14%)	86 (86%)	100 (100%)

Pulmonary function testing (PFT) showed a positive bronchodilator response in 76% of cases overall, and in 16% of ABPA patients (Table 4).

**Table 4:** ABPA vs PFT PFT result with post-bronchodilator increase of >12% and >200 mL in FEV1 was observed in 16% of ABPA cases. The association was not statistically significant (p = 0.49). Data are presented as numbers and percentages: n (%) Abbreviations: PFT, pulmonary function test; FEV1, forced expiratory volume in one second; ABPA, allergic bronchopulmonary aspergillosis

PFT Result	ABPA Present	ABPA Absent	Total
Positive	12 (16%)	64 (84%)	76 (100%)
Negative	2 (8%)	22 (92%)	24 (100%)
Total	14 (100%)	86 (100%)	100 (100%)

**Figure 2:**
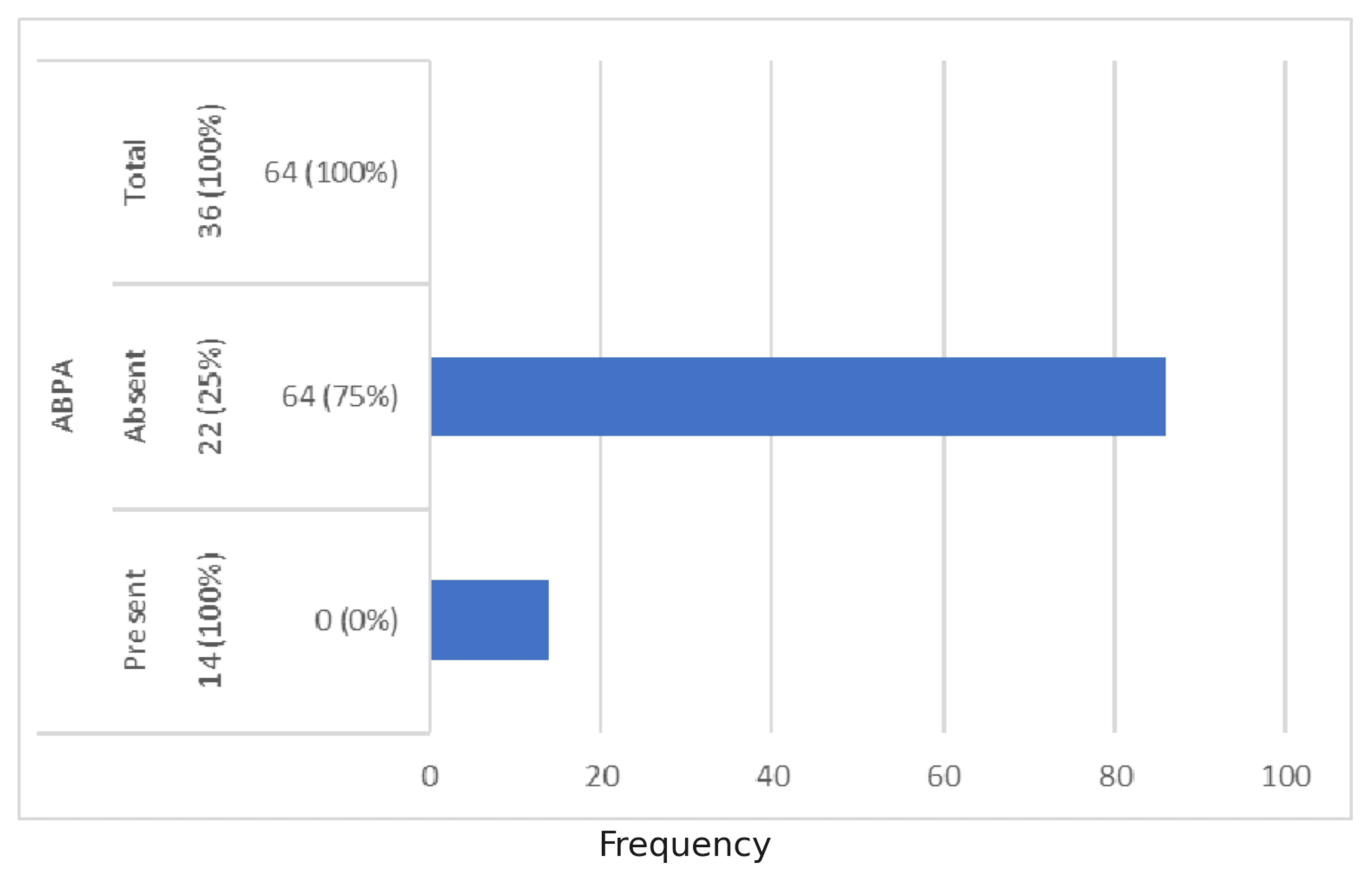
ABPA vs Serum Total IgE Serum total IgE >1000 ng/ml was observed in 39% of allergic bronchopulmonary aspergillosis (ABPA) cases, with a sensitivity of 100% and specificity of 74.42%.

Serum total IgE levels >1000 ng/mL were elevated in all ABPA patients, resulting in a sensitivity of 100% and specificity of 74.42% (Figure 2).

**Figure 3:**
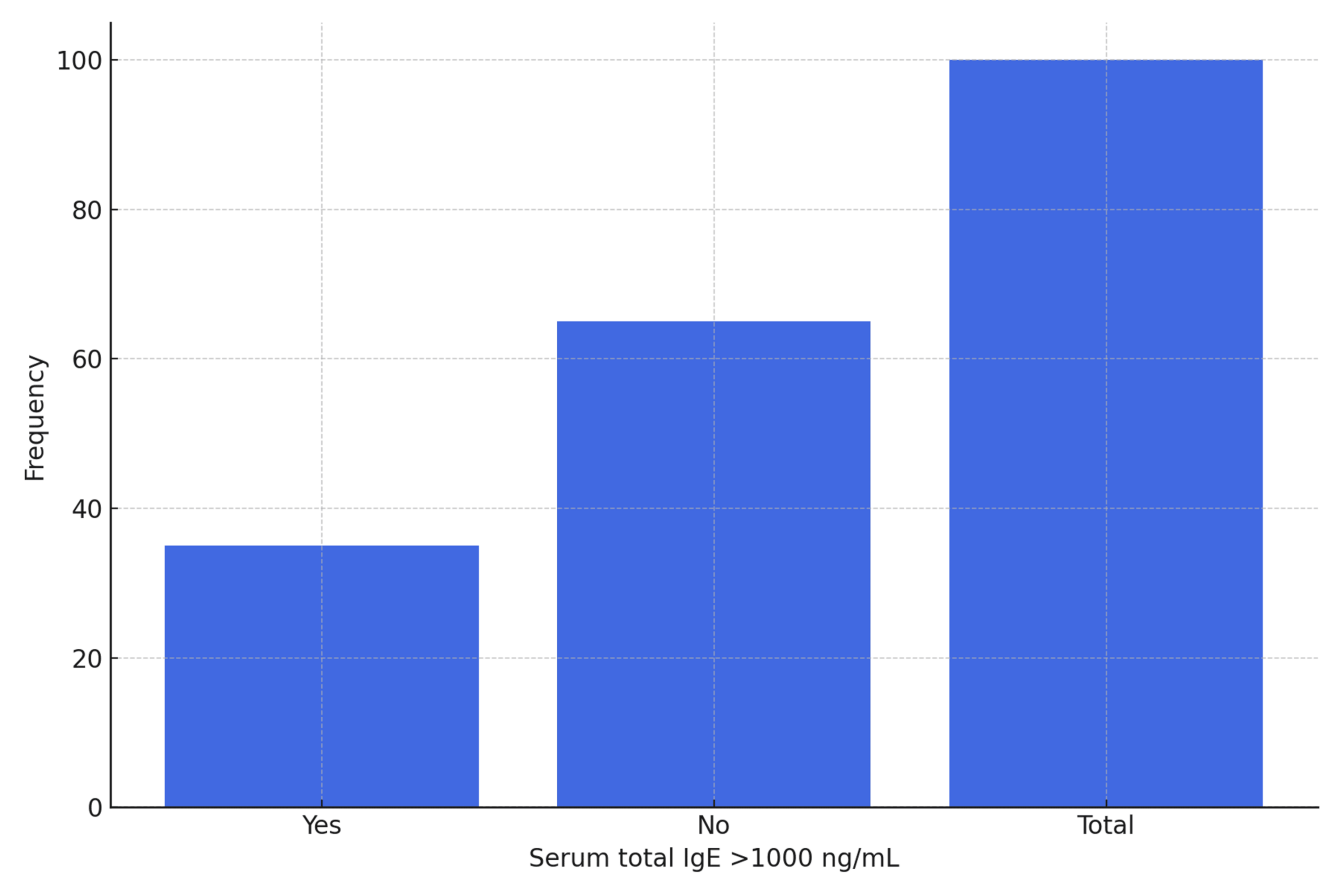
Serum Total IgE >1000 ng/ml Serum total IgE >1000 ng/ml was observed in 36% of the study population.

In the overall study population, serum total IgE >1000 ng/mL was observed in 36% of patients (Figure 3).

Clinical history and comorbidities

**Figure 4:**
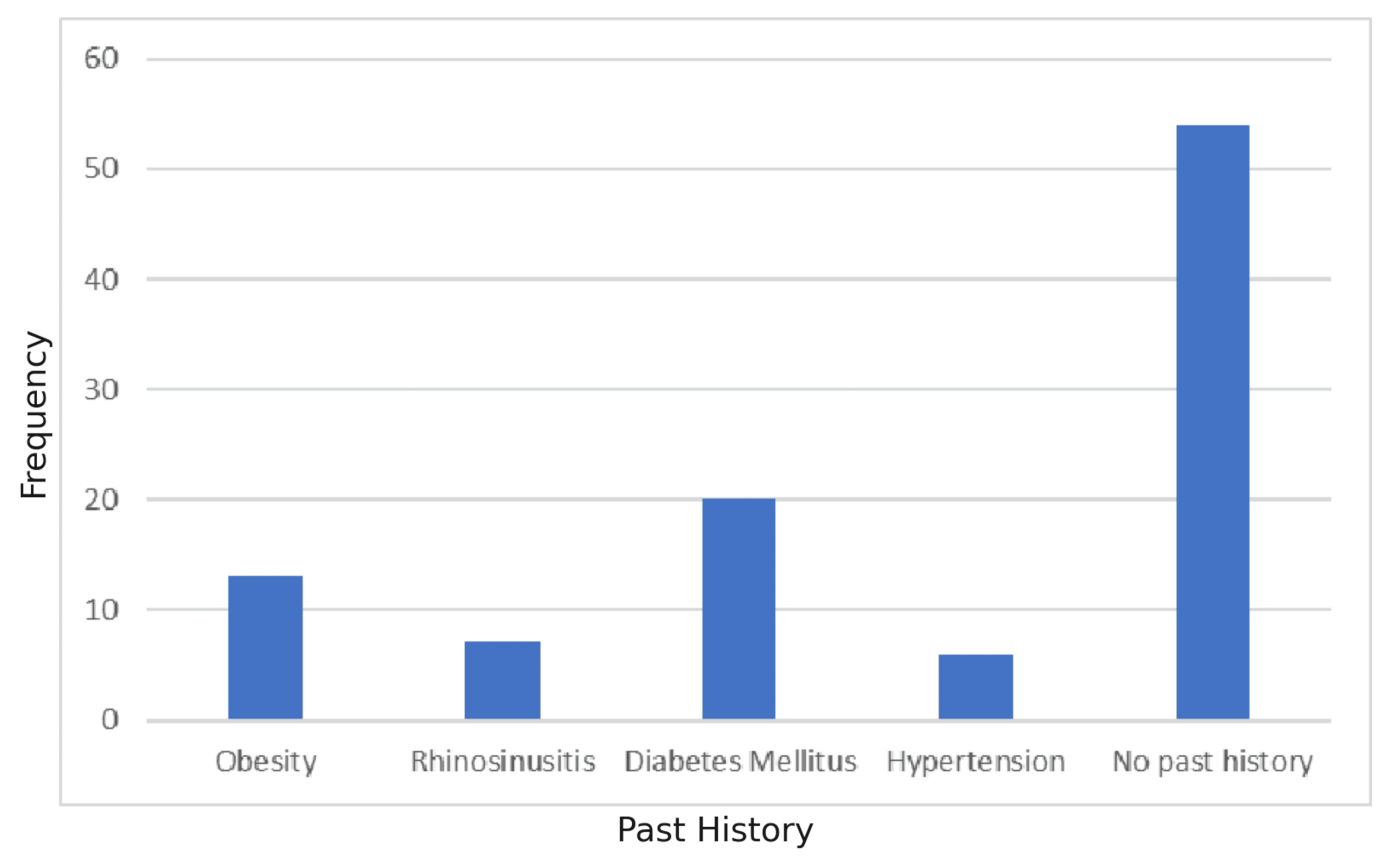
Past History Diabetes mellitus was the most common comorbidity (20%), followed by obesity (13%), rhinosinusitis (7%), and hypertension (6%).

A history of addictions was prevalent, with Dennie’s addiction reported in 31%, tobacco chewing in 24%, alcohol consumption in 23%, smoking in 13%, and *mishri *(smokeless tobacco) use in 9% of the study population. Additionally, a positive family history of bronchial asthma was noted in 43% of participants. Regarding comorbid conditions, diabetes mellitus was observed in 20% of patients, followed by obesity in 13%, rhinosinusitis in 7%, and hypertension in 6% (Figure 4). Dyspnea was the most commonly reported clinical symptom (64%), followed by cough (46%), chest tightness (18%), wheeze (28%), fever (12%), and hemoptysis (7%).

## Discussion

ABPA is a condition in which a patient develops an allergic reaction to *Aspergillus *spores. It predominantly affects individuals with asthma, cystic fibrosis, or bronchiectasis. Approximately 1-5% of adult asthmatics and chronic obstructive pulmonary disease (COPD) patients may develop ABPA during their lifetime. The clinical presentation of ABPA often mimics atypical asthma, necessitating a high index of suspicion for diagnosis [1].

Age group

In the present study, the most common age group among the study population was 21-30 years (27%), followed by 18-20 years (26%), 31-40 years (23%), and 51-60 years (14%), with a mean age of 30.45 years. These findings are consistent with a study by Kalaiyarasan et al. in which the majority of ABPA patients belonged to the 25-45 years age group (mean, 35.33 years) and no sex predilection was noted [8]. The mean age in various studies ranged from 31.2 to 36.25 years [6-10]. Although ABPA can occur at any age, the majority of cases are seen between 20 and 40 years. ABPA has also been reported in children [9] and even in infants [11].

Gender

In this study, there was a male predominance (69%) compared to females (31%). This aligns with findings by Kalaiyarasan et al., who reported 77% of ABPA cases in males [8]. Similarly, Shubhra Jain et al. reported a male predominance (60%), and Nousheen Iqbal et al. [9, 10] found that 69.6% of their ABPA cases were male. 

Socioeconomic status

Most participants in the current study belonged to lower socioeconomic classes: lower (36%), upper lower (24%), lower middle (21%), and upper middle (12%). Socioeconomic status was inferred indirectly through school types, with private schools representing upper socioeconomic classes and government schools representing lower classes. A study by Amir et al. made similar observations, noting a higher prevalence of asthma among children attending private schools [11].

Comorbidities

Diabetes mellitus (20%) was the most common comorbidity in the study population, followed by obesity (13%), rhinosinusitis (7%), and hypertension (6%). Obesity is recognized as a risk factor for asthma, especially in women [12-16]. A meta-analysis reported asthma risk ratios of 1.20 for overweight and 1.43 for obese men; for women, the risks were 1.25 and 1.78, respectively [17]. US and Norwegian studies have similarly shown elevated odds ratios ranging from 1.29-1.84 for obese males and 1.55-1.96 for obese females [18-22].

Type 1 diabetes is thought to result from autoimmune processes possibly triggered by viruses, while type 2 diabetes is associated with obesity, insulin resistance, and systemic inflammation [21, 23-26]. A large Kaiser Permanente study found that the incidence of asthma was 0.16 per 1,000 person-years in non-diabetics and 0.41 in diabetics [27]. This association was confirmed in Danish twin studies [28] and hospitalized patient data [25]. Proposed mechanisms include inflammatory cytokines, effects on insulin sensitivity, and the use of systemic glucocorticoids in severe asthma, which may increase the risk of type 2 diabetes [29].

Chest X-ray infiltrates

Chest X-ray infiltrates were seen in 62% of the total study population and in 23% of ABPA cases, with a sensitivity of 100% and specificity of 44.19%. This aligns with the study by R. Prasad et al., who reported chest X-ray infiltrates in 69.1% of ABPA patients [24].

Pulmonary function test

Pulmonary function test (PFT) (defined as a post-bronchodilator increase of >12% and >200 mL in FEV1) was positive in 76% of participants and in 16% of ABPA cases, with a sensitivity of 85.71% and specificity of 25.58%. Similar findings were observed in Stevenson and Szczeklik [12].

Serum total IgE

Serum total IgE levels >1000 ng/mL were seen in 36% of the total population and in 100% of ABPA cases, with a sensitivity of 100% and specificity of 74.42%. Kalaiyarasan et al. also reported that IgE levels are mildly elevated in allergic asthma but markedly elevated in ABPA, typically exceeding 417 IU/mL (1000 ng/mL) [8]. However, serum total IgE, like absolute eosinophil count (AEC), is neither sensitive nor specific for diagnosing ABPA, as levels may vary with disease stage and corticosteroid use.

HRCT thorax

In this study, HRCT confirmed ABPA in 23% of participants. ABPA represents a hypersensitivity reaction to *Aspergillus *species, leading to airway inflammation and asthma [8]. About 1-2% of asthma patients develop ABPA [9, 10]. Though considered rare in India [11], Previous studies have also reported variable prevalence rates of ABPA among asthma patients, with Stevenson and Szczeklik documenting a prevalence of 16% and Cohn et al. reporting 7.5%, highlighting differences that may be attributed to study design, diagnostic criteria, and population characteristics [12, 13].

Radiologically, ABPA can show transient or permanent shadows. Transient lesions (e.g., perihilar infiltrates, air-fluid levels, or consolidations) typically resolve with or without corticosteroids and are not pathognomonic. These are referred to as “fleeting shadows” due to their migratory nature [16, 17]. The hallmark radiologic feature of ABPA is central bronchiectasis with preserved peripheral bronchi, seen as “string of pearls” or “signet ring” on HRCT. High-attenuation mucus is considered a specific indicator of ABPA [18-20,30].

Limitations

This study has several limitations that may affect the validity and generalizability of its findings. Firstly, its retrospective design relied on hospital records, which may contain incomplete documentation, inaccuracies, or misclassification of cases. Secondly, there is potential for selection bias, as only patients who were evaluated and diagnosed were included, while undiagnosed or subclinical cases may have been missed. Thirdly, no formal sample size calculation was performed, and the relatively small cohort reduces the statistical power of subgroup analyses. Additionally, potential confounding variables such as comorbidities, prior corticosteroid use, or environmental exposures were not controlled for, which could influence both asthma severity and ABPA prevalence.

Temporal ambiguity regarding the onset of symptoms and timing of investigations may also affect interpretation. The diagnostic criteria for ABPA, while based on established guidelines, may have varied over the study period as thresholds evolved, introducing potential misclassification bias. Finally, the single-centre design limits generalizability, as the findings may not reflect broader or more diverse populations. These limitations highlight the need for larger, multicentric prospective studies using standardized diagnostic protocols and multivariate analyses to validate and extend our findings.

## Conclusions

This study revealed a 14% prevalence of ABPA among bronchial asthma patients, highlighting the importance of screening in this subgroup. The most common clinical manifestation was dyspnea, followed by cough, wheeze, and chest tightness. ABPA was observed across all asthma severities, with a higher proportion in mild asthma (21%), though the association was not statistically significant. Key diagnostic indicators included serum total IgE >1000 ng/mL, which showed the highest sensitivity (100%) and a specificity of 74.42%, supporting its strong diagnostic value. Chest X-ray infiltrates and HRCT thorax findings also contributed to ABPA detection, albeit with lower specificity. These findings underscore the need for a high index of suspicion and comprehensive diagnostic workup for ABPA in asthma patients, particularly in tertiary care settings.
